# Immediate Results of Resection of Stomach

**Published:** 1914-08

**Authors:** 


**Immediate Results of Resection of Stomach.** S. Miyake, Sei-I-Kwai Med. Jour., May 10, 1914, states that to date, 346 cancer cases have been admitted to his hospital of the Kyushu University. 319 were subjected to some form of operation. Excision was done on 132 cases, on 116 by the author himself. of the 116, 82 recovered, the mortality rate being 29.9%. Four years ago, his mortality was 23 (32.8%) in a series of 70. The recent cases show 11 deaths out of 46, a trifle less than 25%. He holds that the apparent superiority of Kocher's method is due to its adaptibility to early cases, while Billroth's method is required for advanced cases.

